# Association between serum level of Vitamin D with autoantibodies expression, disease activity (SLEDAI) and bone mineral density (BMD) in patients with Systemic Lupus Erythematosus (SLE)

**DOI:** 10.1186/ar3624

**Published:** 2012-02-09

**Authors:** Handono Kalim, Singgih Wahono, Putra Suryana BP, Lenny Puspitasari, Fajar Hadi Wijayanto, Kusworini Handono

**Affiliations:** 1 Rheumato-Immunology Division, Department of Internal Medicine, Brawijaya University, Malang, Indonesia; 2 Department of Clinical Pathology Faculty of Medicine, Brawijaya University, Malang, Indonesia

## Backround

Vitamin D defficiency has been reported to have negative association with clinical manifestation and disease activity of SLE [1,2]. Vit D has an important role in the pathogenesis of SLE [3] and it is necessary to give vit D supplementation to the patients [4]. The objective of our study was to determine the association between serum vitamin D level with auto antibodies expression, disease activity and bone mineral density in SLE patients.

## Patients and methods

55 female patients with SLE were recruited from Clinic of Rheumato-Immunology, Saiful Anwar Hospital, Malang, Indonesia. Mean age of the patients 31.12 years (12-64 yo) with duration of illness 18,4 months (2-54 mo). Serum vitamin (25 (OH)D3 level was assayed using ELISA method (Cusabio, normal value>30 ng/mL). Anti ds-DNA and Anti Cardiolipin antibodies were assayed using ELISA method (Diagnostic Automation, Inc, USA). Disease activity assessed by SLE disease activity index (SLEDAI) and BMD was assessed by bone densitometry using DEXA. Association between variables (serum vitamin D and autoantibodies level, BMD and SLEDAI) were analyzed using Spearman correlation.

## Result

The mean of serum 25(OH)D3 level was 22.80 ± 16,23 ng/mL.14 patients (25.5%) had vitamin D deficiency (<10 ng/mL), 34 patients (61.8%) had vitamin D insufficiency (10-30 ng/mL), and 7 patients (14.7%) had normal vitamin D levels. There were significant difference level of anti-dsDNA antibodies (112.46 vs 267.13 U/ml; p < 0.05) and IgM ACA (16.40 vs 29.7 IU/ml; p < 0,05) in patients with vitamin D insufficiency and vitamin D defisiency. Serum level of 25(OH)D3 were negatively related with level of anti-dsDNA and IgM ACA (r: - 0, and r: - 0,72 respectively). The mean of SLEDAI was 15,0 + 10.46. Serum vitamin D levels were inversely correlated with SLEDAI (*R=*-0.319, *p*<0.05). Normal BMD at lumbal spine found in 21 (38.2%) patients. 26 patients (47.3%) were osteopenia, and 8 (14.5%) patients were osteoporosis. At femoral neck, 25 (45.5%) patients had normal BMD, 23(41.8%) patients were osteopenia, 7 (12.7%) patients were osteoporosis. There were no significant correlation between vitamin D level and BMD at lumbal spine (*p = 0,531*) and at femoral neck (*p = 0,175*).

## Conclusion

A large proportion of SLE patients had low vitamin D levels. There were positive association between vit D level and autoantibodies expression in SLE and negative association between serum vitamin D levels with SLEDAI. No association was found between serum vit D level and BMD.
